# Scrotal Dermatofibrosarcoma Protuberans in a Patient with Schizophrenia: A Case Report and Review of the Literature

**DOI:** 10.7759/cureus.54369

**Published:** 2024-02-17

**Authors:** Leah Ashton, Gabrielle R Yankelevich, Kyler W Perry, Robert L Grubb

**Affiliations:** 1 Department of Urology, Medical University of South Carolina, Charleston, USA

**Keywords:** scrotectomy, social determinants of health, schizophrenia and other psychotic disorders, schizophrenia, scrotal mass, dermatofibrosarcoma protuberans

## Abstract

Dermatofibrosarcoma protuberans (DFSP) is a rare spindle cell soft tissue sarcoma of the dermis and subcutaneous tissue. We present the fourth case of scrotal DFSP in the literature, identified in a 32-year-old male with schizophrenia. Wide surgical excision and radical orchiectomy were performed revealing an uninvolved testicle and DFSP of the scrotum. A unique challenge to this case was concurrent aortic dissection and schizophrenia. Social determinants of health are associated with delay in presentation and poor appointment compliance in patients with schizophrenia. Ultimately, DFSP of the scrotum is an extremely rare condition with this presentation being only the fourth report in the literature. It is important to document these unique cases to establish differential diagnoses and optimize management.

## Introduction

Soft tissue sarcomas arise from mesenchymal cells of connective tissue and are infrequent tumors, accounting for less than 1% of adult malignancies [1,2]. Dermatofibrosarcoma protuberans (DFSP) is a rare spindle cell soft tissue sarcoma of the dermis and subcutaneous tissue that involves only 1% of all soft tissue sarcomas [3]. DFSP often presents as a slow-growing painless plaque, which, over time, may progress to a nodule or ulcerate, becoming painful. Most often, DFSP can be found on the trunk (50-60%), upper limb (25%), or head/neck (10-15%) [2]. There are only three previously reported cases of DFSP of the scrotum, and we present, to our knowledge, the fourth case in the literature [4-6].

## Case presentation

**Figure 1:**
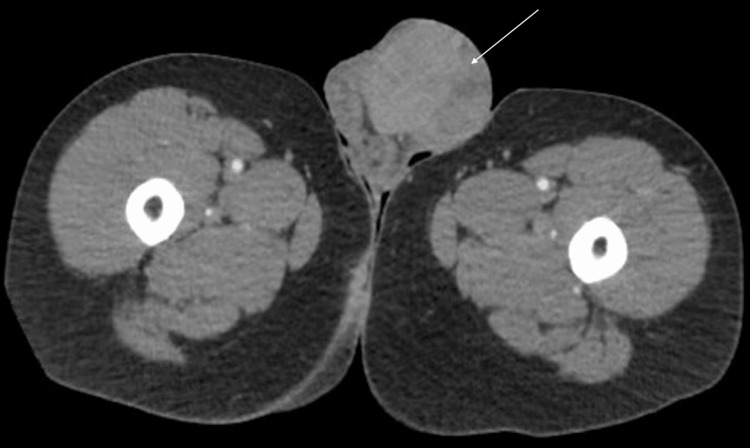
CT abdomen pelvis through the thigh demonstrating a large, left-sided scrotal mass (white arrow)

**Figure 2:**
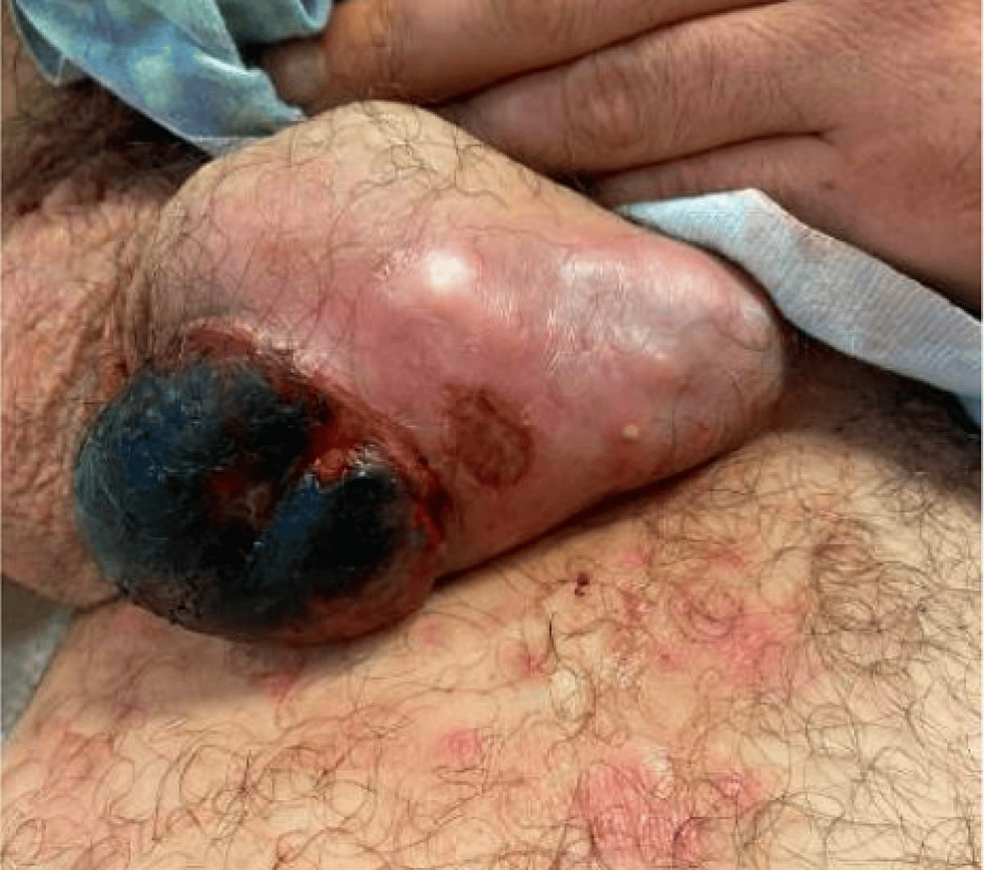
Physical examination of the scrotum with a left-sided necrotic mass

A 32-year-old male with a past medical history of intellectual disability, type 2 bipolar disorder, and schizophrenia presented to a community hospital with severe scrotal discomfort for one to two weeks. The patient had a necrotic left scrotal mass for which a CT abdomen/pelvis was obtained. The scan showed a large left scrotal mass measuring 7.2 x 6.6 x 7.4 cm (Figure 1) concerning for testicular neoplasm and an incidental type B aortic dissection from the distal to the left subclavian artery to the common iliac arteries bilaterally. The patient was transferred to our center on beta-blockers for the management of his aortic dissection, and urology was consulted for the scrotal mass. The genitourinary exam showed a large necrotic area on the left hemi-scrotum with the cord and testicle hard to palpation (Figure 2). The right testicle was soft and without lesions. There was no inguinal lymphadenopathy found on examination.

**Figure 3:**
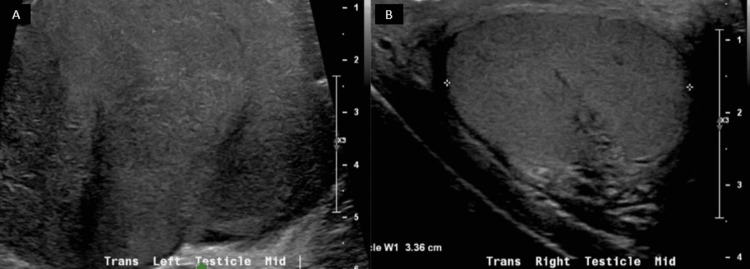
Scrotal ultrasound showing a lobulated, irregular left testicle (A) and normal right testicle (B)

Testicular cancer markers were obtained, which were all noted as within normal limits: AFP (alpha-fetoprotein) < 2.0, b-hCG (beta-human chorionic gonadotropin) < 2.42, and LDH (lactate dehydrogenase) of 203.0. A testicular ultrasound was obtained to ensure the right testicle did not have a synchronous tumor. The ultrasound showed a normal right testicle with a lobulated, heterogenous left testicular mass (Figures 3A-3B) concerning for testicular neoplasm.

The patient was evaluated by vascular surgery for several days, and it was determined that the aortic dissection was chronic. Once the patient was determined to be stable from a vascular standpoint, he was taken to the operating room for a left radical orchiectomy via inguinal approach and partial scrotectomy closed primarily. Surgical exploration revealed that the large, necrotic left scrotal mass was not actually contiguous with the left testicle but had an extension into the spermatic cord. Frozen sections of the scrotum intra-operatively revealed a spindle cell neoplasm. The final pathology was a 9 cm grade 1 DFSP of the scrotum with negative margins, confirmed with cytogenetic fluorescence in situ hybridization (FISH). The testicle was uninvolved.

## Discussion

There are only three previously reported cases of DFSP of the scrotum in the literature, and we present, to our knowledge, the fourth case. Given that DFSP is a very rare disease, the diagnosis hinges on pathology, typically with the use of immunohistochemistry. Radiographic findings are nonspecific for this pathology.

Histologically, DFSP presents as bland spindle cells arranged in storiform or whorled patterns with uniform spindle cells containing elongated nuclei and minimal cytoplasm [2,7]. Differentials before staining can include other spindle cell tumors such as solitary fibrous tumors and undifferentiated spindle cell sarcomas [7]. DFSP has strong staining of CD34 (cluster of differentiation 34) and apolipoprotein D, which can differentiate it from other pathologies [7].

**Figure 4:**
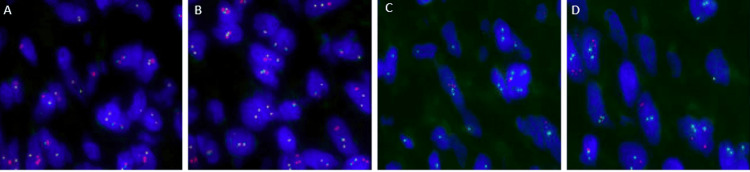
Cytogenic FISH with detection of PDGFB gene rearrangement (A-D) Immunofluorescent areas on cytogenic FISH indicate positive PDGFB gene rearrangement expression. A-D are four different slices of the specimen revealing positive PDGFB expression. FISH: Fluorescence in situ hybridization; PDGFB: Platelet-derived growth factor subunit B

Though DFSP has a characteristic histological appearance, as this is an exceedingly rare diagnosis, further testing was conducted on the specimen for confirmation. Cytogenetic FISH was performed, which demonstrated 68% abnormal nuclei with normal < 9.0% (Figure 4). The signal pattern showed deletion of 5’ with retention of 3’ portion of platelet-derived growth factor subunit B (PDGFB), known to be active in oncogenic fusions, which is indicative of unbalanced PDGFB gene rearrangement. PDGFB rearrangement is characteristic of dermatofibrosarcoma [8-10]. Chromosomal microarray analysis demonstrated a partial gain of 17q (breakpoint in COL1A1 (collagen type 1 alpha 1), involving 5' region) and partial gain of 22q (breakpoint in PDGFB, involving 3' region) in 95% of cells, as well as loss of 7q in less than 5% of cells. Breakpoints in COL1A1 and PDGFB strongly suggest unbalanced rearrangement of chromosomes 17 and 22 resulting in COL1A1/PDGFB fusion, which has been reported to be present in 85-96% of patients with DFSP [11,12].

Standard management of DFSP involves wide local excision (WLE), Mohs micrographic surgery (MMS), or amputation, depending on the lesion's location. Regardless of the method used, it is important to obtain tumor-free margins. Local recurrence is of great concern in this population, occurring in 20-50% of patients. The median time from WLE to recurrence is 32-48 months; therefore, close follow-up is necessary to mitigate risk [13,14]. In cases of unresectable lesions or metastasis, several studies have shown that radiation can be utilized [14].

Prognosis after surgical resection has a 10-year recurrence-free survival rate of 76% [14]. Local recurrences are higher with positive margins, with frequencies of 20-50% [14]. There is limited data on the length of time for surveillance, but studies suggest interval imaging every six months for the first five years and then annually afterward [14]. This patient was presented to the multi-disciplinary tumor board with recommendations for post-operative abdominopelvic magnetic resonance imaging (MRI) and surveillance MRIs every six to 12 months.

Lastly, it is important to discuss the social determinants of health that led to the challenges of this case. The patient had an intellectual disability and schizophrenia, which likely contributed to the delay in seeking medical care. Given the rarity of this pathology, research regarding the relationship between schizophrenia and DFSP has not been conducted, however, a large meta-analysis found that patients with psychotic disorders had a higher incidence of testicular cancer (relative risk 1.27) and 22% higher odds of metastasis at diagnosis compared to those without psychotic disorders [15]. Additionally, patients with schizophrenia had high rates of appointment non-adherence, with the prevalence of a missed first appointment after hospitalization being over 30% [16-17]. This patient is now three months from surgery and unfortunately has not presented to follow-up appointments despite multiple attempts to reach the patient and family members. Finally, the aortic dissection was a unique challenge and unrelated to the presenting illness. Ultimately, it is important to involve a multidisciplinary team to help mitigate barriers to healthcare; in this case, urology, vascular surgery, medicine, psychiatry, and social work were consulted.

## Conclusions

DFSP of the scrotum is an extremely rare condition with only three prior reports in the literature. It is important to document these unique cases to establish differential diagnoses and optimize management. Immunohistochemistry can be an important tool to further differentiate tumors and subsequently help guide treatment.
